# Genome-Wide Association Studies of Salt Tolerance at Seed Germination and Seedling Stages in *Brassica napus*

**DOI:** 10.3389/fpls.2021.772708

**Published:** 2022-01-05

**Authors:** Guofang Zhang, Jinzhi Zhou, Yan Peng, Zengdong Tan, Long Li, Liangqian Yu, Cheng Jin, Shuai Fang, Shaoping Lu, Liang Guo, Xuan Yao

**Affiliations:** ^1^National Key Laboratory of Crop Genetic Improvement, Huazhong Agricultural University, Wuhan, China; ^2^Hubei Hongshan Laboratory, Wuhan, China

**Keywords:** salt stress, germination stage, seedling stage, GWAS, *Brassica napus*

## Abstract

Most crops are sensitive to salt stress, but their degree of susceptibility varies among species and cultivars. In order to understand the salt stress adaptability of *Brassica napus* to salt stress, we collected the phenotypic data of 505 *B. napus* accessions at the germination stage under 150 or 215 mM sodium chloride (NaCl) and at the seedling stage under 215 mM NaCl. Genome-wide association studies (GWAS) of 16 salt tolerance coefficients (STCs) were applied to investigate the genetic basis of salt stress tolerance of *B. napus*. In this study, we mapped 31 salts stress-related QTLs and identified 177 and 228 candidate genes related to salt stress tolerance were detected at germination and seedling stages, respectively. Overexpression of two candidate genes, *BnCKX5* and *BnERF3* overexpression, were found to increase the sensitivity to salt and mannitol stresses at the germination stage. This study demonstrated that it is a feasible method to dissect the genetic basis of salt stress tolerance at germination and seedling stages in *B. napus* by GWAS, which provides valuable loci for improving the salt stress tolerance of *B. napus*. Moreover, these candidate genes are rich genetic resources for the following exploration of molecular mechanisms in adaptation to salt stress in *B. napus*.

## Introduction

Salt stress is a leading cause of inhibition of crop growth and development in the world (Munns, 2005; Morton et al., 2019). At present, about 10% area of China is arable land is salt-affected (Song and Liu, 2017). Although many methods have been used to improve the high yield of crops under salt stress, it is also a challenge of maintaining the world food supplies (Hickey et al., 2019; Tyerman and Munns, 2019). Understanding the genetic and molecular mechanisms for enhancing salt tolerance is an important step toward improving the agricultural productivity of crops (Patishtan et al., 2018).

Salt stress mainly includes ion stress and osmotic stress (Zhu, 2002; Julkowska and Testerink, 2015). Short-term salt stress can make the plants immediately appear the phenomenon of wilting dehydration and slow down the growth speed of the new leaves slow down. However, long-term salt stress can cause ion poisoning and a high concentration of ions in the old leaves, leading to falling off of leaves. Crops have different abilities to resist salt stress. When the level of salt stress is greater than the resistance of plants, owing to cell membrane damage, ion imbalance, lipid peroxidation, reactive oxygen species production and metabolic disorders, etc., the damage of high osmotic pressure will restrict the normal growth and development of plants (Bedard and Krause, 2007; Munns and Tester, 2008). Moreover, Salt stress is mainly regulated by hormones and ion transporters in plants, which form a complex genetic regulatory network (Zhu, 2001b,^2016^; Khan and Hakeem, 2013).

The harm of salt stress is also attributed to inhibition of moisture absorption and ions poisoning and their interference with the uptake of mineral nutrients based on a decrease in osmotic potential (Khan and Weber, 2006; Mba et al., 2007). HKT1 encodes a sodium ion (Na^+^) transporter and it contributes to salt tolerance in plants (Apse et al., 1999; Brini et al., 2007). The SOS signaling pathway was involved in Na^+^ extrusion (Ma et al., 2019). The *sos* mutants show hypersensitivity to salt stress in *Arabidopsis* (Zhu, 2001a; Shi et al., 2002), whereas overexpression of *AtSOS1*, *AtSOS2*, and *AtSOS3*-overexpression can significantly improve salt resistance in plants (Zhu, 2001a,^2016^). *OsSKC1* was mapped for root and shoot Na^+^/potassium ion (K^+^) concentration and transportation in the shoots and roots controlling rice salt tolerance (Lin et al., 2004). Two Na^+^/H^+^ antiporters, *AtNHX1* and *AtNHX2*, localized on the tonoplast membrane. They are expressed in roots and leaves, and selectively transports Na^+^ into the vacuole. *nhx1 and nhx2* mutants showed decreased sensitivity to salt stress in *Arabidopsis* (Brini et al., 2007; Barragan Borrero et al., 2012).

Salt stress usually makes a significant inhibitory effect on seed germination and seedling growth of crops, which plays an important role in stable stand establishment and yield of crops (Rehman et al., 2000). Previous studies have shown that germination rate, shoot length, root length, and aboveground dry weight at the seed germination stage are extremely sensitive to salt stress in crops (Gomes-Filho et al., 2008; Munns et al., 2012; Yu et al., 2018). Moreover, plant height, root length, Na^+^/K^+^ ratio, relative electrical conductivity, aboveground fresh weight, and dry weight can also be affected by salt stress during the seedling period in rice (Campbell et al., 2015), wheat, barley (Fan et al., 2016), tomato (Campbell et al., 2015), *Arabidopsis* (Awlia et al., 2016), and *B. napus* (Hou et al., 2017; Lang et al., 2017). To date, only a few studies have focused on the gene function related to salt stress tolerance of *B. napus*. The *AtNHX1* overexpression could significantly improve the salt tolerance of *B. napus* (Zhang et al., 2001). The *BnaABF2* could significantly enhance salt tolerance in transgenic *Arabidopsis* (Zhao et al., 2016). The 582 transcription factors and 438 transporter genes have been identified by a comparative transcriptome analysis under salt stress (Yong et al., 2014). With the development of sequencing technology, genome-wide association study (GWAS) is a quick tool to map QTLs of complex traits for crop genetic improvement (Pearson and Manolio, 2008; De et al., 2014). *Bna.SCO1*, *Bna.ARR4*, and *Bna.ATE1* was identified at the germination stage under salt stress by GWAS in 248 *B. napus* accessions using the 60k SNP array (Hatzig et al., 2015). In addition, 38 promising candidate genes associated with salt tolerance were also identified by GWAS in 368 Brassica accessions using a 60k SNP array (Wan et al., 2017). A total of 62 QTLs for aboveground dry weight and Na^+^/K^+^ ratio traits were identified in 85 *B. napus* inbred lines (Yong et al., 2015). GWAS could improve efficiency and cost savings to parse the genetic basis based on abundant genetic variation and prediction precision (Huang et al., 2012). *OsMADS31* could be a down-regulated expression and was identified by GWAS to be a candidate gene related to salt stress response at the germination stage of rice (Yu et al., 2018). Although a great number of QTLs and candidate genes have been identified by previous studies in *B. napus*, no candidate genes are functionally validated in response to salt stress and molecular mechanisms underlying the regulation of salt tolerance have not been elucidated in *B. napus*.

To study the genetic basis of the adaptation of *B. napus* to salt stress, GWAS of salinity-stress responsive traits at seed germination and seedling stages in the 505 *B. napus* accessions was employed to map QTLs related to the salt stress responses. Here we totally identified 177 and 228 candidate genes controlling 16 salt stress coefficients (STCs) of *B. napus* subjected to salt stress at seed germination and seedling stages, respectively. It should be noted that many previously unreported genes were discovered to be involved in salt stress responses. This study will help us unveil the mechanism of salt stress adaptation and provide a valuable reference for the genetic improvement of salt tolerance in *B. napus*.

## Materials and Methods

### Materials

A collection consisting of 505 natural accessions of *B. napus* (Supplementary Table 1; Tang et al., 2021) was used to investigate growth in normal and salt stress conditions at seed germination and seedling stages (Supplementary Table 2).

### Trait Measurement at Seed Germination and Seedling Stages

The experiment involved 100 uniform seeds which were treated by three treatment groups including normal (CK), low salt (150 mM sodium chloride [NaCl]), and high salt stress (215 mM NaCl) and put into a Petri dish (Φ 9 cm, Guangzhou Jiete Biological Filtration Co., Ltd., Wuhan, China) with double layers of filter paper (Φ 9 cm, General Electric Biotech Hangzhou Co., Ltd., Wuhan, China). Then, we added 10 ml deionized water (CK), 150 mM (T1), and 215 mM (T2) NaCl solution into the petri dish with a peri dish cover in the growth room for 3 days, 7 days, and 2 weeks to record germination potential (FYS), germination rate (FYL), shoot length (SL), and root length (RL), respectively (Supplementary Figure 1). All experiments were carried out in a greenhouse with 25/16°C day/night temperature under a 16/8 h of light/darkness photoperiod and 50–60% RH (Long et al., 2015).

For measurements at the seedling stage, the plants were grown in ten rounds of experiments, and each round contained about 50 accessions. We designed 2 treatment groups including normal (CK) and high salt stress (215 mM NaCl solution) conditions with 6 biological repeats per line (Supplementary Figure 2). In order to guarantee reliability, we firstly selected 25–30 uniform and healthy seeds and then put the seeds into the yarn net in hydroponic culture for a generation. After 10 days, the uniform seedlings were selected and transferred to a 10 L black box covered by a black plate with Hoagland nutrient solution (60 cm length × 40 cm width × 10 cm depth) according to a completely randomized block design. After these plants were cultivated in the nutrient solution culture for about 2 weeks, we added 10 L Hoagland nutrient solution (CK) and Hoagland nutrient solution containing 215 mM (T2) NaCl solution into the black box, respectively, and the nutrient solution was changed once a week. The seedlings were treated for 2 weeks in the hydroponic culture. Hoagland nutrient solution consists of the following nutrients: 4 mM KNO_3_, 1 mM MgSO_4_, 4 mM Ca (NO_3_)_2_, 1 mM NH_4_H_2_PO_4_, 1 mM (NH_4_)_2_HPO_4_, 1 mM NaCl, 41.2 μM Na_2_-EDTA, 12.5 μM H_3_BO_3_, 0.39 μM CuSO_4_, 1.59 μM MnSO_4_, 1 μM ZnCl_2_, and 0.5 μM NaMoO_4_, and pH of the solution was adjusted to 5.8 with 0.1 M KOH (Sinopharm Chemical Reagent Co., Ltd., Wuhan, China) (Monirifar and Barghi, 2009; Wan et al., 2017).

### Determination of Growth Indexes

#### Growth Indexes

After a 2-week salt treatment, some traits, such as plant height (PH), RL, aboveground dry weight (ADW), and root dry weight (RDW) were measured. Materials were harvested and dried at 85°C for at least 72 h to be weighed (Lv et al., 2016; Yu et al., 2017).

#### Leaf Area

Leaf area was measured by a high-throughput leaf area meter developed by the phenotyping platform of phenotype platform center of Huazhong Agricultural University (Yang et al., 2015).

#### Leaf Chlorophyll Content

Leaf chlorophyll content was determined using the Chlorophyll Meter SPAD-502plus (Spectrum Technologies Inc., Hangzhou, China) under salt stress and normal conditions. The fully-expanded penultimate leaf was used for the measurement. Each leaf was measured three times at different positions avoiding the veins, and the average of the three readings was recorded at different time points under salt stress conditions (Kang et al., 2019).

#### Determination of Proline

Proline was an important component of plants in response to salt stress. L-proline concentration was determined using a standard curve. About 0.1 g of leaves were homogenized in 1.5 ml of 3% sulfosalicylic acid (Sinopharm Chemical Reagent Co., Ltd., Wuhan, China) and the residue was removed by centrifugation at 3,000 rpm/min for 5 min. 100 μl of the extract was reacted with 2 ml glacial acetic acid and 2 ml acid ninhydrin (Sinopharm Chemical Reagent Co., Ltd., Wuhan, China) (1.25 g acid ninhydrin warmed in 30 ml glacial acetic acid and 20 ml 6 M phosphoric acid until dissolved) for 1 h at 100°C and the reaction was then terminated in an ice bath. The reaction mixture was extracted with 1 ml toluene. The vortex shocked for 30 s and rested 10 min. The chromophore-containing toluene was warmed to room temperature and its optical density was measured at 520 nm *via* an ultra-microporous plate (MPP) spectrophotometer (BioTek Epoch, United States) (Bates et al., 1973; Khedr et al., 2015).

#### Determination of Malondialdehyde

We weighed approximately 0.1 g of leaf tissue per plant, to which we added 2 ml of 10% trichloroacetic acid (TCA, Sinopharm Chemical Reagent Co., Ltd., Wuhan, China) solution followed by the addition of to achieve a total volume of 3 ml. The samples have subsequently centrifuged the sample for 10 min, at 4°C at 4,000 rpm. We transferred 2 ml solution of the supernatant into new tubes and added 2 ml of distilled water and 2 ml of 0.6% thiobarbituric acid (TBA, Sinopharm Chemical Reagent Co., Ltd., Wuhan, China) solution, respectively. After a 15-min reaction in boiling water for a 15 min reaction, after which the solution was centrifuged and cooled to room temperature. Finally, we measured the malondialdehyde (MDA) content at wavelengths of 450, 532, and 600 nm *via* an ultra-microporous plate (MPP) spectrophotometer (BioTek Epoch, United States) (Hong et al., 2005; Wang et al., 2006).

#### Relative Electrical Conductivity

Relative electrical conductivity measurements were performed as previously described with minor modifications (Lv et al., 2016). One fully expanded functional leaf from normal plants was cut into segments of similar sizes by a leaf blade sampler and immersed in 8 ml of double-distilled water in a 10 ml tube for 24 h at room temperature with continual shaking at 100 rpm, and then we calculated the parameter (S1) by a conductivity meter (Model DDS-IIA, Shanghai Leici Instrument, Inc., Shanghai, China). The rest of the solution was placed into a water bath at 100°C for 10 min. After the sample was cooled down to room temperature, the conductivity value was measured (S2) (Model DDS-IIA, Shanghai Leici Instrument, Inc., Shanghai, China). The value of REC was calculated as the ratio of S1 to S2.

#### Statistical Analysis

Student’s *t*-test was performed to calculate the statistically significant differences between data sets. For each accession, the absolute values of all traits were obtained and the average of 6 replicates was counted under each condition. The salt tolerance coefficient was calculated by STC = T/WT. We used the scale package of R language to analyze data standardization. Phenotypic data were analyzed using the stat.desc package of R language.

#### Genetic, Treatment Effect, and Heritability Analysis

To determine the variance components, we used mixed-effect ANOVA of avo package of R language including the genetic effect (G_effect), treatment effect (E_effect), and interaction effect of genetic and treatment effect (G & E effect). Broad sense heritability (*H_2_b*) was calculated for all traits as follows: *H_2_b* = σ*^2^_*G*_/(*σ*^2^_*G*_* + σ*^2^_*GE*_/n* + σ*^2^_*e*_/nr)* where σ*^2^_*G*_* was the genotypic variance, σ*^2^_*e*_* was the error variance, σ^2^_GE_ is the variance of the genotype by environment interaction and r is the number of replications with R language. The estimates of σ*^2^_*G*_* and σ*^2^_*e*_* were analyzed by the ANOVA using the lmer function in the lme4 package in the R environment. All statistical analyses were performed in R language (Chen et al., 2014).

#### Correlation Analysis

The correlation coefficient was calculated using the corr.test of psych package and plotted using the corrplot package of R language.

#### Genome-Wide Association Analysis

The 505 *B. napus* accessions were collected to construct this association panel (Tang et al., 2021). The high-quality clean reads data was used by the BWA (version 0.75) software (Li and Durbin, 2009). The reference genome came from “Brassica version 4.1” (‘Darmor-bzh’) genome^1^. We adopted a mixed-model approach using the factorial spectrally transformed linear mixed model with 6,448,413 SNPs (MAF > 0.05). We also performed GWAS using FaST-LMM software models (Listgarten et al., 2013).

#### Identification of Candidate Genes and Polymorphisms

The suggestive and significant *P*-value thresholds of the entire population were 1.0e−06. All SNPs that exceeded the significance criterion were assessed for the location of candidate genes. If coding regions were present, the potential impact on the protein of each SNP was subsequently determined using the “Allele finder” facility Gene Ontology (GO) analysis. The candidate genes with differential gene expression ratios between the control treatment and stress treatment (R ≥ 2 or ≤ 0.5) (Zhang et al., 2019) were identified within 200 kb upstream or downstream of the lead SNP (Tang et al., 2021).

#### RNA-Seq

Total RNA was extracted with TRIzol reagent (Invitrogen Life Technologies, Wuhan, China) from the leaves of 4-week-old plants grown under normal conditions and 385 mM NaCl in a growth chamber set at 25 C, a 16/8 h light/dark and 50–60% relative humidity. The RNA was dissolved in DEPC water and measured with a spectrophotometer (NanoDrop, Beijing, China, 2000). The purified RNA was sequenced with GenoSeq (high-throughput phenotyping://www.genoseq.cn/) using an Illumina HiSeq platform center of Huazhong Agricultural University in paired-end 2 × 150 bp mode, and approximately 10 Gb of clean reads were generated for each sample. We finally calculated the normalized transcripts per million (TPM) values. (Supplementary Table 15).

#### Generation of Overexpression Transgenic Plants

The coding DNA sequences of BnaA02g05340D (*BnCKX5*, Primer_F: CGGGGTACCGCCACCATGATAGCTTACATAGA GCCATACT, Primer_R: GCTCTAGATCATTTGTCGTCGTC GTCCTTGTAGTCCATAGGAGCCCTATTGAAAATCTTTTG) and BnaA06g02670D (*BnERF3*, Primer_F: CGGGGTACC GCCACCATGAGGAGAGGTAGAGTCTCT, Primer_R: GCTC TAGATTATTTGTCGTCGTCGTCCTTGTAGTCCATGAGAC GTAGATCGGTGCATAT, Tsingke Biological Technology Wuhan Co., Ltd., Wuhan, China) were cloned from accession X182 (97097), and the cloned fragments were subsequently ligated into pCAMBIA-1300s using the *Kpn*I and *Xba*I restriction enzymes. Cloning primers and sequencing primers are from Tsingke Biological Technology Wuhan Co., Ltd Wuhan, China. The accession, specifically Westar, was used as the transgenic receptor material. Agrobacterium tumefaciens-mediated transformation was performed in *B. napus* as previously described (Dai et al., 2020).

#### Salt and Mannitol Treatments on Overexpression Transgenic Plants

A total of 50 seeds of *OE-BnCKX5*, *OE-BnERF3*, and Westar (WT) were sown into Petri dishes with covers on them and grown in a greenhouse at 25/16°C day/night temperature under a 16/8 h light/dark photoperiod and 50–60% RH. *OE-BnCKX5* lines and WT were treated by 0, 50, 100, 125, 150, and 200 mM NaCl and mannitol for 9 days, respectively. *OE-BnERF3* lines and WT were treated by 0, 50, 100, 125, 150, 175, 200, and 225 mM NaCl and mannitol for 9 days, respectively. Germination rate, stem length, and root length were determined after the salt and mannitol treatments.

## Results

### Phenotypic Variation in 505 *Brassica napus* Accessions for Salt Tolerance Traits at Germination and Seedling Stages

To study the response of the 505 *B. napus* accessions to salt stress, we analyzed the related traits at the germination stage under 0 mM, 150 mM (T1), and 215 mM NaCl (T2), and at the seedling stage under 0 mM and 215 mM NaCl (T2). The germination potential (FYS_CK, FYS_T1, and FYS_T2), germination rate (FYL_CK, FYL_T1, and FYL_T2), shoot length (GSL_CK and GSL_T1), and root length (GRL_CK and GRL_T1) at the germination stage were measured (Supplementary Tables 2, 3). At the seedling stage, we obtained traits under the normal and salt stress conditions, such as plant height (PH_CK and PH_T2), root length (RL_CK and RL_T2), root dry weight (RDW_CK and RDW_T2), aboveground dry weight (ADW_CK and ADW_T2), total dry weight (TDW_CK and TDW_T2), leaf area (LA_CK and LA_T2), malondialdehyde content (MDA_CK and MDA_T2), proline content (Proline_CK and Proline_T2), chlorophyll content under (SPAD_CK and SPAD_T2), and relative electrical conductivity (REC_CK and REC_T2) (Supplementary Tables 2, 4). In order to better reflect salt stress responses, we focused on the salt tolerance coefficient (STC) calculated as the ratio of trait value under salt stress condition to that under normal condition, which was represented by the suffix type “trait_R1” under low salt stress and “trait_R2” under high salt stress (Supplementary Table 5; Guo et al., 2018). Descriptive statistics is a good description of phenotypic variation in 505 *B. napus* accessions under normal and salt stress conditions. We calculated the descriptive statistics through “Mean,” “Max,” “Min,” “SD,” “SE,” and “CV” for the mean values (Supplementary Table 6). We found that most characters were, on average, reduced by more than 25% (CV,%) under salt stress (Supplementary Table 6). Most traits of the accessions show significant variations under salt stress conditions. Interestingly, large variations in germination potential (FYS) and germination rate (FYL) were observed among the accessions under salt stress, whereas FYS and FYL showed small variations under the normal conditions at the germination stage (Supplementary Table 6).

### Frequency Distribution and Correlation Analysis Among All Traits Under Salt Stress

**FIGURE 1:**
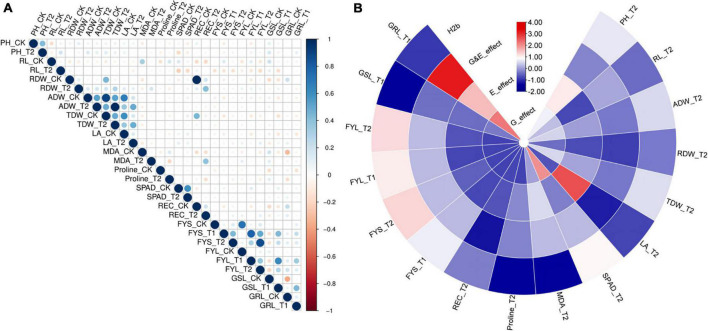
Evaluation of correlation coefficient, heritability, and genetic & treatment effect of all traits under normal and salt stress conditions at germination and seedling stages. **(A)** Heat map showing Pearson’s correlations (r) among all traits of the 505 *B. napus* accessions assessed under normal and salt stress conditions (CK, control; T1, 150 mM NaCl; and T2, 215 mM NaCl) at germination and seedling stages in the greenhouse. **(B)** Heat map showing Broad-sense heritability, genetic, and treatment effect. H2b, broad-sense heritability; E_effect, treatment effect; G_effect, genetic effect and G&E_effcet; interaction of genetic and treatment effects. Germination stage: FYS, germination potential; FYL, germination rate; SL, shoot length and GRL, shoot length. Seedling stage: PH, plant height; RL, root length; RDW, root dry weight; TDW, total dry weight; LA, leaf area; MDA, Malondialdehyde content; SPAD, chlorophyll content; Proline, proline content and REC, Relative electrical conductivity.

We also performed normal distribution detection on the original value (Supplementary Table 7) and the relative value (STCs) for GWAS (Supplementary Table 8), respectively. Except for germination potential (FYS) and germination rate (FYL), the rest of the traits were found in normal and lognormal distributions. The non-normal distribution for FYS and FYL may be attributed to the significant phenotypic differences among these accessions. Correlation analysis for all traits at germination and seedling stages was calculated and presented in Supplementary Table 9. A total of 10 traits at the seedling stage and 4 traits at the germination stage showed significant correlations with r, which ranged from 0.001 to 0.994 between each other (Figure 1A, Supplementary Table 9, and Supplementary Figures 3–5). It is worth noting that the RDW_CK was strongly correlated with the TDW_CK and REC_CK (*r* ≥ 0.4). The RL_CK was strongly correlated with the MDA_CK (*r* ≥ 0.3). The RDW_T2 was strongly correlated with the REC_T2 and TDW_T2 (*r* ≥ 0.3). The ADW_T2 was strongly correlated with the ADW_CK, TDW_CK, TDW_T2, LA_CK, and LA_T2 (*r* ≥ 0.3). The TDW_CK was strongly correlated with the TDW_T2, LA_CK, and REC_CK (*r* ≥ 0.4). Moreover, a strong positive correlation between normal and salt stress conditions was observed for PH, GDW, TDW, and SPAD, and their correlations were from 0.426 to 0.599. By comparing the growth dynamics of normal and salt-treated plants, the positive correlations were in accordance with observation between the traits related to salt stress.

### Heritability, Genetic, and Treatment Effect Analysis

Heritability is a key factor for marking decisions in crop breeding and it is crucial to make an effective design for the breeding scheme (Chen et al., 2014). We also calculated the broad heritability (*H_2_b*), which was presented in Supplementary Table 10. As for these parameters, variation between accessions was determined for a large part by the genotype, corresponding to low to high heritabilities (0.002 < *H_2_b* < 0.66) (Figure 1B and Supplementary Table 10). We found that the *H_2_b* of these traits was more than 0.5 including plant height (PH), ground dry weight (ADB), total dry weight (TDW), and chlorophyll content (SPAD). Compared with the control, we always chose significantly genetic and treatment effects of these traits under the different salt treatments using the AVOVA in the R environment (*P* ≤ 0.05). In addition, the genetic effect and treatment effect (G_effect and E_effect, *P* ≤ 0.05) could significantly represent the genotype effect and environment effect, which were significant for all traits (Figure 1B and Supplementary Table 10). These results showed that normal growth and growth response to salt stress are dynamic traits that are determined for a large part by the genetic effect.

### Genome-Wide Association Studies Revealed QTLs Which Were Associated With Salt Stress in *Brassica napus* and Prediction of Candidate Genes Related to Salt Stress

**FIGURE 2:**
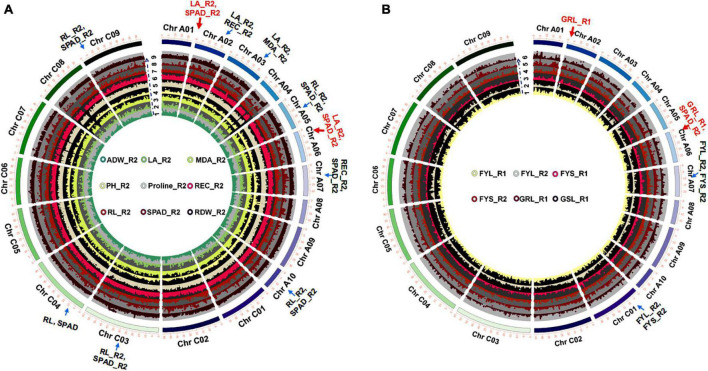
GWAS of STCs at germination and seedling stages. **(A)** Circle Manhattan plots with the co-localized SNPs of multiple traits at the seedling stage (*P*-value ≤ 1e-2; arrows represent sites of co-located SNPs). 1: ADW_R2, the ratio of aboveground dry weight between normal condition and 215 mM NaCl; 2: LA_R2, the ratio of leaf area between normal condition and 215 mM NaCl; 3: MDA_R2, the ratio of MDA content between normal condition and 215 mM NaCl; 4: PH_R2, the ratio of plant height between normal condition and 215 mM NaCl; 5: Proline_R2, the ratio of proline content between normal condition and 215 mM NaCl; 6: REC_R2, the ratio of relative electrolyte leakage between normal condition and 215 mM NaCl; 7: RL_R2, the ratio of root length between normal condition and 215 mM NaCl; 8: SPAD_R2, the ratio of chlorophyll content between normal condition and 215 mM NaCl; 9: RDW_R2, the ratio of root dry weight between normal condition and 215 mM NaCl. **(B)** Circle Manhattan plots with the co-localized SNPs of multiple traits at the germination stage (*P-*value ≤ 1e-2; arrows represent sites of co-located SNPs). 1: FYL_R1, the ratio of germination rate between normal condition and 150 mM NaCl; 2: FYL_R2, the ratio of germination rate between normal condition and 215 mM NaCl; 3: FYS_R1, the ratio of germination potential between normal condition and 150 mM NaCl; 4: FYS_R2, the ratio of germination potential between normal condition and 215 mM NaCl; 5: SL_R1, the ratio of shoot length between normal condition and 150 mM NaCl; and 6: RL_T1, the ratio of root length between normal condition and 150 mM NaCl.

Salt tolerance coefficient (STC) was usually described as an effective salt stress indicator. 16 STCs were performed using the Fast-LMM for GWAS (Listgarten et al., 2013; Figures 2A,B and Supplementary Table 5). The results showed that 5,410 (∼0.08% of in total) strongly associated SNPs [−log(*P*) ≥ 6] (Supplementary Table 11) were chosen to further map the QTLs with the largest effects. There were some co-localized SNPs between leaf area (LA_R2), chlorophyll content (SPAD_R2), relative electrical conductivity (REC_R2), and malondialdehyde (MDA_R2) distributed on Chromosome A02 and A03. In addition, some SNPs were co-localized between chlorophyll content (SPAD_R2) and root length (RL_R2), leaf area (LA_R2), and relative electrical conductivity (REC_R2) distributed on Chromosome A02, A05, A06, and A10 at the seedling stage (Figure 2A and Supplementary Table 12). Some co-localized SNPs between FYL_R2 and FYS_R2 were also found to locate on Chromosome A07 and C01 at the germination stage (Supplementary Table 12 and Figure 2B). Some of these significant SNPs were in linkage disequilibrium and therefore considered to associate with the same causal genes or were assigned to the same QTL.

The candidate genes were selected within 200 kb upstream or downstream of a significant SNP (Supplementary Tables 13, 14). By differential expression analysis comparing normal and treatment conditions (the ratio ≥ 2 or ≤ 0.5) (Zhang et al., 2019), we finally identified 177 candidate genes at the germination stage (Supplementary Table 13) and 228 candidate genes (Supplementary Table 14) at the seedling stage, respectively. Based on our RNA-seq analysis, we also found that these candidate genes were significantly induced by salt stress. Transcription levels of some candidate genes assayed by RNA-seq were shown as an example (Supplementary Tables 14, 15 and Supplementary Figure 6), which was consistent with the previous transcriptome analysis^2^ (Zhang et al., 2019). Most of the candidate genes were found to be related to cellular processes, metabolic processes, biological regulation, signaling, and response to stimulus (Supplementary Figure 7). These results emphasized that complex genetic mechanisms of salt stress responses are regulated by multiple genes in both normal and salt stress conditions at germination and seedling stages.

### *BnCKX5* Overexpression Increased the Sensitivity to Salt and Mannitol Stresses at the Germination Stage

**FIGURE 3:**
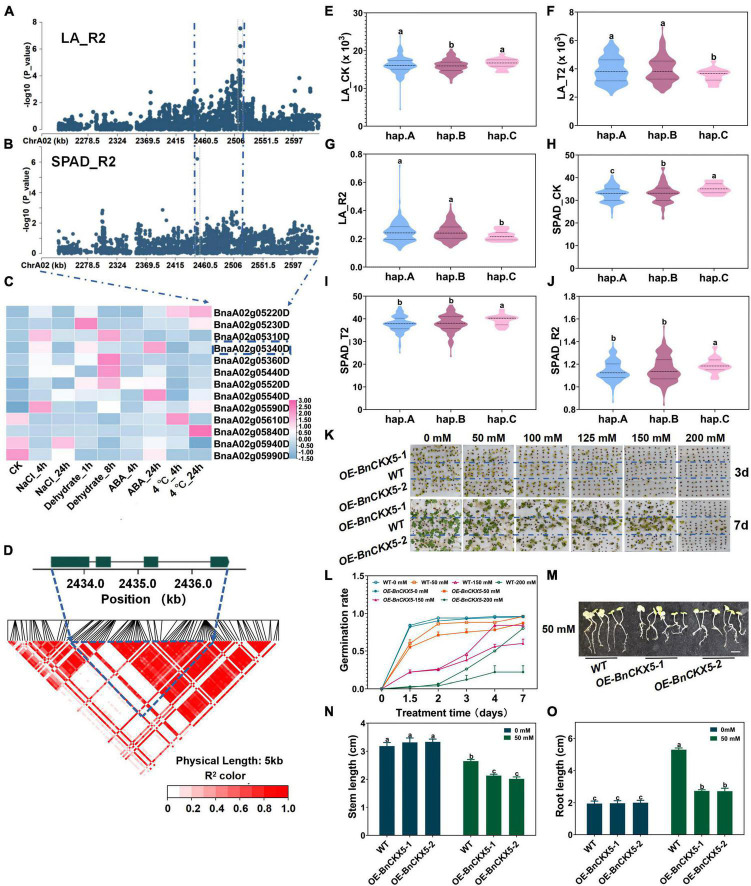
Gene location and function research of *BnCKX5* under salt stress. **(A)** Zoom-in of the Manhattan plot on ChrA02 with co-localized locus. These points represented the log-transformed *P*-values for variants from GWAS of root length (RL_R1) at the germination stage. **(B)** Zoom-in of the Manhattan plot on ChrA02 with co-localized locus. These points represented the log-transformed *P*-values for variants from GWAS of chlorophyll content (SAPD_R2) at the seedling stage. **(C)** Gene expression of candidate genes identified within 200kb upstream or downstream of the associated SNPs under 200 mM NaCl, 25 μM ABA or 4°C low-temperature stress for 4 and 24h and dehydration condition for 1h and 8h in *B. napus* cultivar Zhong Shuang 11 (‘ZS11’). **(D)** LD heatmap within 1kb upstream or downstream of *BnCKX5*. **(E–G)** Boxplots of haplotypes for the absolute and relative value of the root length (RL) at germination stage (RL_CK, **E**; RL_T1, **F**; and RL_R1, **G**). Values were means ± SD and different letters indicate differences at *P* ≤ 0.05 using two-way ANOVA. **(H–J)** Boxplots of haplotypes for the absolute and relative value of different stress conditions and control with the chlorophyll content (SPAD) at seedling stage (SPAD_CK, **H**; SPAD_T2, **I**; and SPAD_R2, **J**). Values were means ± SD (*n* = 6 replicates) and different letters indicate differences at *P* ≤ 0.05 using two-way ANOVA. **(K,L)** Comparison of the germination rate (FYL, **K**,**L**) of wild type (Westar) and *BnCKX5* overexpression (*OE-BnCKX5*) under control and salt (0, 50, 100, 125, 150, 175, 200, 225, and 250 mM NaCl) conditions. Values were means ± SD (*n* = 2 replicates). **(M–O)** Comparison of the shoot length (SL, **N**) and root length (RL, **O**) of wild type (Westar) and *BnCKX5* overexpression (*OE-BnCKX5*) under control and salt (0, 50, 100, 125, 150, 175, 200, 225, and 250 mM NaCl, **M**) conditions. Scale bar = 1.5 cm. Values were means ± SD (*n* = 6 replicates) and different letters indicate differences at *P* ≤ 0.05 using two-way ANOVA.

There are 36 genes on ChrA02 within 200 kb upstream and downstream of the candidate SNPs, BnvaA0202514802 and BnvaA0202514804 of LA_R2, BnvaA0202449329 of SPAD_R2 and BnvaA0201847086 of RL_R1 (Figures 3A,B and Supplementary Table 14). Moreover, among these genes, BnaA02g05340D, whose expression was found to be significantly induced by salt stress and ABA treatment (Figure 3C, Supplementary Figures 6, 15, and Supplementary Table 17) (see text footnote 1) (Zhang et al., 2019), have not yet been reported to be related to salt stress in *B*. *napus*. Thus, we focused on the BnaA02g05340D for further study. BnaA02g05340D named *BnCKX5* is a homolog of *Arabidopsis CKX5* belonging to *CKXs* subfamily VII, which encodes a cytokinin dehydrogenase and plays a vital role in maintaining cytokinin homeostasis (Ma et al., 2016). However, whether *CKX5* plays a role in plant salt tolerance remains unknown (Liu et al., 2018). *BnCKX5* consists of three introns and four exons with pairwise LD correlations, which presents rich SNP sequence variations leading to amino acids changes (*r*^2^ > 0.5) (Figure 3D and Supplementary Table 18). The haplotypes of *BnCKX5* were grouped into 3 haplotypes including hap.A, hap.B, and hap.C types. The absolute and relative values of leaf area (LA) in the Hap.C type accessions were significantly less than that in hap.A and hap.B under salt stress (Figures 3E–G and Supplementary Table 19), while the absolute and relative values of chlorophyll content (SPAD) in the hap.C type accessions were significantly higher than that in hap.A and hap.B type accessions under normal and salt stress conditions (Figures 3H–J and Supplementary Table 19). Therefore, *BnCKX5* was considered as a candidate gene for salt stress response and showed a rich SNPs variation among 505 *B. napus* accessions. The identified extreme haplotypes related to salt tolerance would be useful for breeding salt-tolerant *B. napus* in the future.

To validate whether *BnCKX5* was involved in response to salt stress, seeds of *BnCKX5*-overexpressing (*OE-BnCKX5*) plants and WT were sowed under the 0, 50, 100, 125, 150, and 200 mmol NaCl for 9 days (Figure 3K and Supplementary Figures 8, 9). Germination rates significantly decreased in the *OE-BnCKX5* lines compared with the WT plants under the salt treatments (Figure 3L). Stem length and root lengths of *OE-BnCKX5* lines were significantly lower than that of WT under 50 mmol NaCl (Figures 3M–O). In addition, seeds of *OE-BnCKX5* and WT have also sowed under 0, 50, 100, 125, 150, and 200 mmol mannitol for 9 days. Germination rate significantly decreased in the *OE-BnCKX5* lines compared with the WT plants under the mannitol treatments (Supplementary Figures 10, 11). These results suggest the *BnCKX5* overexpression increases the sensitivity to salt and mannitol stresses at the germination stage. However, the detailed molecular mechanism mediating the adverse effects of salt and mannitol stresses at the germination stage through *BnCKX5* remains to be further investigated.

### *BnERF3* Overexpression Increased the Sensitivity to Salt and Mannitol Stresses at the Germination Stage

**FIGURE 4:**
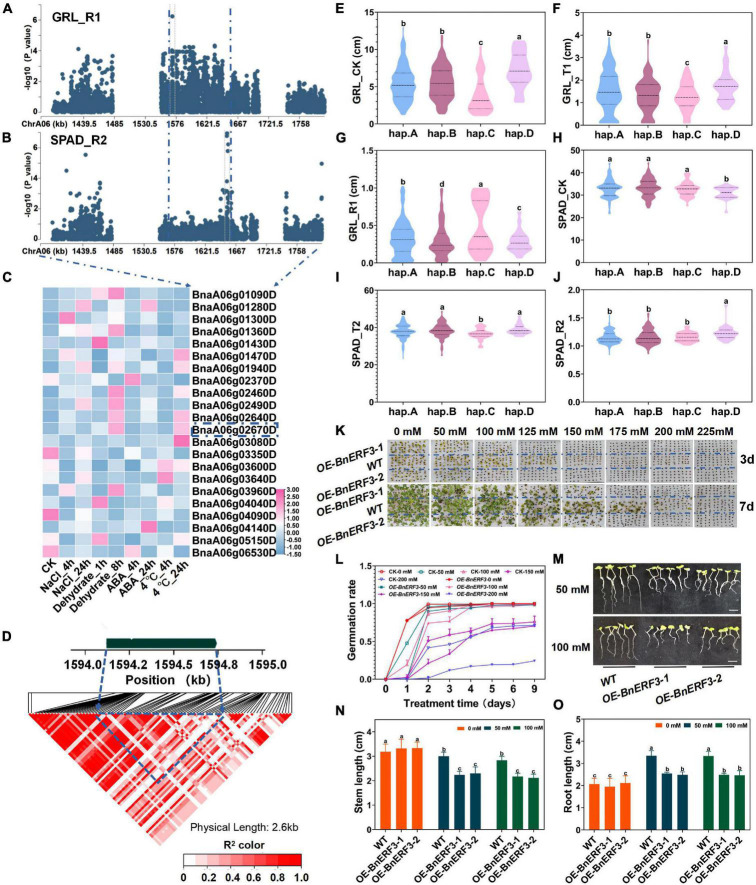
Gene location and function research of *BnERF3* under salt stress. **(A)** Zoom-in of the Manhattan plot on ChrA06 with co-localized locus. These points represented the log-transformed *P*-values for variants from GWAS of root length (RL_R1) at the germination stage. **(B)** Zoom-in of the Manhattan plot on ChrA06 with co-localized locus. These points represented the log-transformed *P*-values for variants from GWAS of chlorophyll content (SAPD_R2) at the seedling stage. **(C)** Gene expression of candidate genes identified within 200kb upstream or downstream of the associated SNPs under 200 mM NaCl, 25 μM ABA or 4°C low-temperature stress for 4 and 24h and dehydration condition for 1 and 8h in *B. napus* cultivar Zhong Shuang 11 (“ZS11”). **(D)** LD heatmap within 1kb upstream or downstream of *BnERF3*. **(E–G)** Boxplots of haplotypes for the absolute and relative value of different stress conditions and control with the root length (RL) at germination stage (RL_CK, **E**; RL_T1, **F**; RL_R1, **G**). Values were means ± SD and different letters indicate differences at *P* ≤ 0.05 using two-way ANOVA. **(H–J)** Boxplots of haplotypes for the absolute and relative value of different stress conditions and control with the chlorophyll content (SPAD) at seedling stage (SPAD_CK, **H**; SPAD_T2, **I**; SPAD_R2, **J**). Values were means ± SD (*n* = 6 replicates) and different letters indicate differences at *P* ≤ 0.05 using two-way ANOVA. **(K,L)** Comparison of the germination rate (FYL, **K,L**) of wild type (Westar) and *BnERF3* overexpression (*OE-BnERF3*) under control and salt (0, 50, 100, 125, 150, 175, 200, 225, and 250 mM NaCl) conditions. Values were means ± SD (*n* = 2 replicates) and different letters indicate differences at *P* ≤ 0.05 using two-way ANOVA. **(M–O)** Comparison of the shoot length (SL, **N**) and root length (RL, **O**) of wild type (Westar) and *BnERF3* overexpression (*OE-BnERF3*) under control and salt (0, 50, 100, 125, 150, 175, 200, 225, and 250 mM NaCl, **M**) conditions. Values were means ± SD (*n* = 2 replicates). Scale bar = 1.5 cm. Values were means ± SD (*n* = 6 replicates) and different letters indicate differences at *P* ≤ 0.05 using two-way ANOVA.

By analyzing SNPs sequence variations of genes, we totally identified 38 genes by SNPs cluster on ChrA06 within 200 kb upstream and downstream of the candidate SNPs, from BnvaA0601466004 to BnvaA0601655665 of SPAD_R2 and BnvaA0601570302 of RL_R1 (Figures 4A,B and Supplementary Table 14). Moreover, among these genes, BnaA06g02670D, whose expression was found to be significantly induced by salt stress and dehydration treatment, has not yet been reported to be related to salt stress in *B*. *napus*. Thus, we focused on the BnaA06g02670D for further study (Figure 4C, Supplementary Figure 6, and Supplementary Tables 15, 17) (see text footnote 1) (Zhang et al., 2019). BnaA06g02670D named *BnERF3* is a homolog of *Arabidopsis ERF3* belonging to *the* AP2/ERF subfamily, which encodes an ethylene response transcription factor and plays a very important role in signal transduction of many adversity stresses. However, whether *ERF3* plays a role in plant salt tolerance remains unknown (Zhang and Huang, 2010). *BnERF3* consists of one exon with pairwise LD correlations, which presents rich SNP sequence variations leading to amino acids changes (*r*^2^ > 0.5) (Figure 4D and Supplementary Table 20). The haplotypes of *BnERF3* were grouped into 4 haplotypes including hap.A, hap.B, hap.C, and hap.D type accessions (Supplementary Table 21). The absolute and relative value of root length (RL) in the Hap.D type accessions was significantly less than that in hap.A, hap.B, and hap.C under salt stress (Figures 4E–G and Supplementary Table 21). However, the absolute and relative values of chlorophyll content (SPAD) in the hap.D type accessions were significantly higher than that in hap.A, hap.B and hap.C type accessions under normal and salt stress conditions (Figures 4H–J and Supplementary Table 21). Therefore, *BnERF3* was considered as a candidate gene for salt stress response and showed a rich SNPs variation among 505 *B. napus* accessions. The identified extreme haplotypes related to salt tolerance would be useful for breeding salt-tolerant *B. napus* in the future.

To examine whether *BnERF3* participates in response to salt stress, seeds of *BnERF3*-overexpressing (*OE-BnERF3*) plants and WT were sowed under the 0, 50, 100, 125, 150, 175, 200, and 225 mmol NaCl for 9 days (Figure 4K and Supplementary Figures 12, 13). Germination rates significantly decreased for the *OE-BnERF3* lines compared with the WT plants under the salt treatment (Figure 4L). The stem length and root length of *OE-BnERF3* were significantly lower than that of WT under 50 mmol NaCl (Figures 4M–O). In addition, seeds of *OE-BnERF3* lines and WT have sowed under 0, 50, 100, 125, 150, 175, 200, and 225 mmol mannitol for 9 days. Germination rates significantly decreased in the *OE-BnERF3* lines compared with the WT plants under the mannitol treatments (Supplementary Figures 14, 15). These results suggest the overexpression of *BnERF3* increases the sensitivity to salt and mannitol stresses at the germination stage. However, the regulation mechanism of *BnERF3* involved in response to salt and mannitol stresses at the germination stage needs further investigation.

## Discussion

Seed germination has significant influences on seedling establishment and yield performance. Germination speed under different stress conditions is a key element of vigorous seeds to measure the resistance of seed germination (Finch-Savage et al., 2010; Hatzig et al., 2015). In this study, we determined the response to salt stress at seed germination and seedling stages in 505 accessions of *B. napus*. Four phenotypic indexes and 10 growth and physiological traits were obtained at seed germination (Supplementary Tables 2, 3) and seedling stages (Supplementary Tables 2, 4), respectively.

The genetic effect and treatment effect (G_effect and E_effect, *P* ≤ 0.05) could better represent the genotype effect and treatment effect, which were significant for all traits under salt stress (Supplementary Table 10). We found that the salt stress could significantly inhibit the plant height, root length, leaf area, aboveground dry weight and total dry weight at the seedling stage, and germination rate, shoot length, and root length at germination stage compared with control. On the contrary, salt stress significantly increased the REC, MDA, and SPAD. The broad-sense heritability could better represent the genotypic effect of all traits ranging from 1.31E-06 (GSL) to 0.664 (SPAD). Smaller plants with lower STCs were observed under salt stress. A positive correlation was observed between normal and salt stress conditions. The correlation coefficients of all traits were calculated at germination and seedling stages. The ADB was significantly correlated with TDW and LA (*p* < 001). The germination potential was positively correlated with germination rate, shoot length, and root length under salt stress (*p* < 0.05), in accordance with the previous studies (Taghvaei et al., 2012; Amlca and Yaldz, 2017). Our findings revealed that the traits of germination were not significantly correlated with the seedling traits, suggesting that the regulatory mechanism of salt stress responses was likely to be different between the germination and seedling stages. These results were consistent with a previous study (Wan et al., 2017).

Two previous two studies have performed GWAS to explore the genetic basis of salt stress responses at the seedling stage in 85 and 368 inbred *B. napus* lines (Yong et al., 2015; Wan et al., 2017). Though many QTLs and candidate genes related to salt stress at the seedling stage were identified, no candidate gene has been functionally validated under salt stress in *B. napus* until now. Using a GWAS approach, we mapped 31 salts stress-related QTLs by GWAS of 16 STCs to investigate the genetic basis of salt stress tolerance of *B. napus* (Supplementary Table 11). However, parts of those SNPs could be false positives to salt stress responses. In addition, the lack of overlapped QTLs can also be the consequence of the weak power to detect the many micro-effect genes, which probably underlie the salt stress responses. Detection power was reduced by the low heritability observed in our salt stress conditions at germination and seedling stages, respectively. A total of 75 co-localized and special SNPs were found by Venn analysis between the two stages (Supplementary Table 12). Therefore, we believe that our method is trustworthy.

In addition, we totally identified 177 candidate genes at the germination stage (Supplementary Table 13) and 228 candidate genes (Supplementary Table 14) at the seedling stage, which are differentially expressed under salt stress, respectively (see text footnote 1) (Zhang et al., 2019). Some candidate genes identified from the GWAS results have been reported to be related to salt stress responses, such as BnaA03g43130D (*RAB*), BnaA05g01580D (*ABI1*), BnaA07g12170D (*ABA1*), BnaA10g29660D (*SOS3*), and BnaC07g25850D (*ABI5*) (Yong et al., 2015). Moreover, we also mapped some candidate genes, which also were identified in a previous study (Huang et al., 2012). BnaA01g02240D and BnaA01g02100D were identified for the salt tolerance index of root length (ST-RL). BnaA05g03980D, BnaA05g05230D, BnaA05g04990D, BnaA07g22240D, and BnaA07g22790D were identified for shoot length (ST-SL). BnaA01g12890D was identified for shoot fresh weight (ST-SFW) (Wan et al., 2017). Therefore, we believed that it was a reliable method to identify candidate genes related to salt stress. Interestingly, some of these candidate genes have not yet been known to be involved in salt stress responses. Additionally, BnaA02g03750D (AT5G17860.1) is a calcium exchanger protein. BnaA06g01090D (AT1G53170.1) belongs to the AP2/ERF subfamily, which encodes an ethylene response factor transcription factor and plays an important role in signal transduction of many adversity stresses (Savitch et al., 2005). BnaC05g00520D (AT1G01490.2) encodes a heavy metal transport/detoxification superfamily protein, which plays a role in ion transportation. BnaA02g07120D and BnaA02g07110D (AT5G59310.1) encode lipid transfer proteins. BnaA02g05360D (AT5G21940.1) encodes a histidine kinase. These identified candidate genes were also found to be differentially expressed under salt, drought stress, or ABA treatment (see text footnote 1) (Zhang et al., 2019).

Additionally, the function of two identified candidate genes by transgenetic verification, which are *BnCKX5* and *BnERF3*, has also not yet been reported to be related to salt stress. BnaA02g05340D (*BnCKX5*) encoding a cytokinin dehydrogenase is involved in the cytokinin metabolic process. Previous studies have discovered that the *ckx3 ckx5* double mutants in *Arabidopsis thaliana* form large inflorescences, floral meristems, and more ovules, thereby increasing the number of kernels per silique (Liu et al., 2018). Four *BnCKX3* and two *BnCKX5* genes were to be regulators of reproductive development in the allotetraploid *B. napus*. *ckx3 ckx5* mutants increased cytokinin concentration and larger and more active inflorescence meristems (Ireen et al., 2020). *PsCKX2* and *PsCKX5* formed citrus dwarf rootstock with a stronger root system with more lateral roots in *Poncirus trifoliata* (Ma et al., 2016). BnaA06g02670D (*BnERF3*) encodes a member of the ethylene response factor subfamily and is an AP2 transcription factor, which negatively regulates the ethylene-activated signaling pathway. *AtERF4* encoding an AP2 transcriptional repressor modulate ethylene and abscisic acid responses in *Arabidopsis* (Zhen et al., 2005). Therefore, we are curious about whether *BnCKX5* and *BnERF3* have functions in the adaptation of *B. napus* to salt stress. Our results revealed that *BnCKX5* and *BnERF3* played positive roles in response to salt and mannitol stresses in *B. napus*. However, more studies are needed to unveil the molecular mechanism of *BnCKX5* and *BnERF3* regulation in response to salt stress. These results suggest that GWAS is employed not only to confirm the presence of allelic differences in known salt tolerance-related genes but also to identify QTLs of which the causal gene is not annotated yet to be involved in salt tolerance. Our study provided an efficient way to reveal the complex genetic architecture of salt stress tolerance to *B. napus*.

## Conclusion

Our results revealed significant natural variation for many phenotypic indexes under salt stress at the germination and seedling stages and rich genetic variation of candidate genes related to salt stress responses. Many traits were correlated to salt stress response, indicating the overlap in salt regulatory networks or correlated responses of these traits to selection. Many candidate genes with unknown function in salt stress responses, such as *BnCKX5* and *BnERF3*, were identified based on differential expression under salt stress by haplotype analyses and genetic transformation. *BnCKX5* and *BnERF3* overexpression were found to increase the sensitivity to salt and mannitol stresses at the germination stage. Additionally, some salt-tolerant and salt-sensitive accessions have been identified as breeding materials for the genetic improvement of salt stress tolerance of *B. napus*. This study provided insights into the genetic architecture of salt tolerance at germination and seedling stages by GWAS and would be useful for genetic improvement of salt tolerance by breeding in *B. napus*.

## Data Availability Statement

The original contributions presented in the study are included in the article/Supplementary Material, further inquiries can be directed to the corresponding author.

## Author Contributions

XY and LG designed the research. GZ, JZ, YP, ZT, LL, LY, CJ, SF, XY, and SL performed the experiments or analyzed the data. GZ, LG, and XY analyzed the data and wrote the manuscript. All authors contributed to the article and approved the submitted version.

## Conflict of Interest

The authors declare that the research was conducted in the absence of any commercial or financial relationships that could be construed as a potential conflict of interest.

## Publisher’s Note

All claims expressed in this article are solely those of the authors and do not necessarily represent those of their affiliated organizations, or those of the publisher, the editors and the reviewers. Any product that may be evaluated in this article, or claim that may be made by its manufacturer, is not guaranteed or endorsed by the publisher.

## Footnotes

1
https://www.genoscope.cns.fr/brassicanapus/data/

2
https://bigd.big.ac.cn/
