# Supplementary material for: Genome-Wide Association Studies of Salt Tolerance at Seed Germination and Seedling Stages in Brassica napus
Source: Front Plant Sci. 2022 Jan 5;12:772708. doi: 10.3389/fpls.2021.772708 (PMC8766642; doi:10.3389/fpls.2021.772708)
Supplement: Supplementary file 2 [file Data_Sheet_2.docx]

**Supplementary Figure**

**
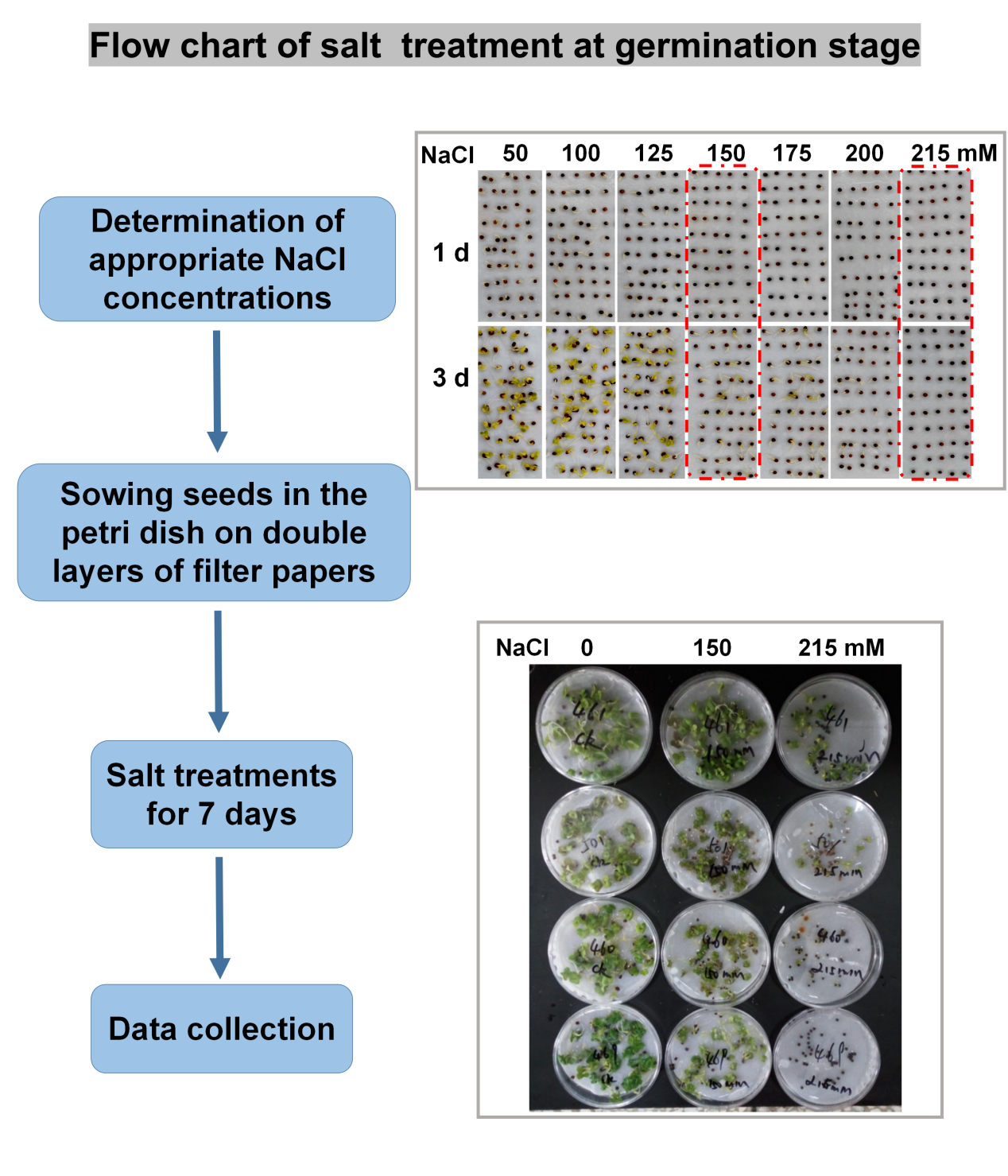
**

**Supplementary Figure S1.** Flow chart of salt treatment at germination stage.


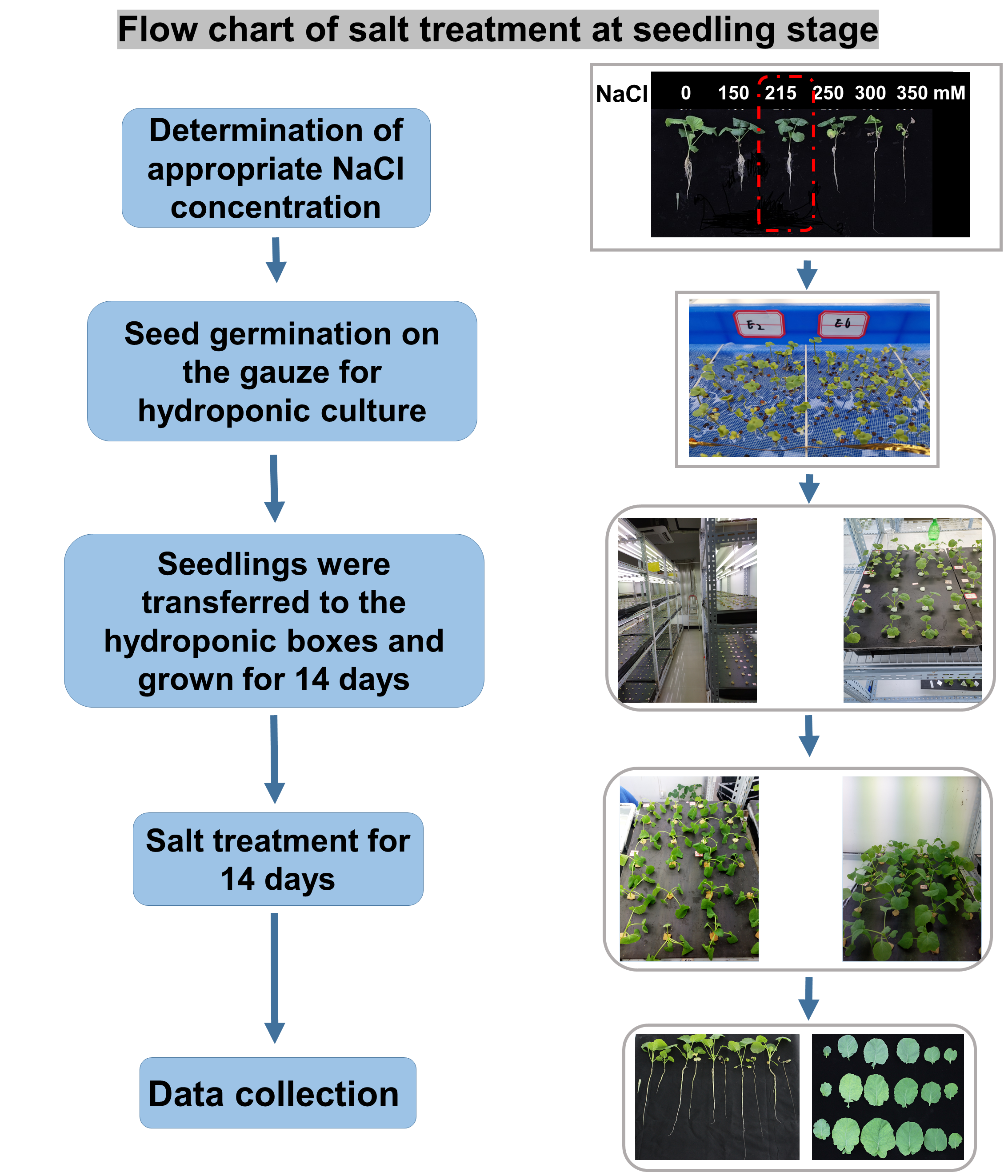


**Supplementary Figure S2.** Flow chart of salt treatment at seedling stage.


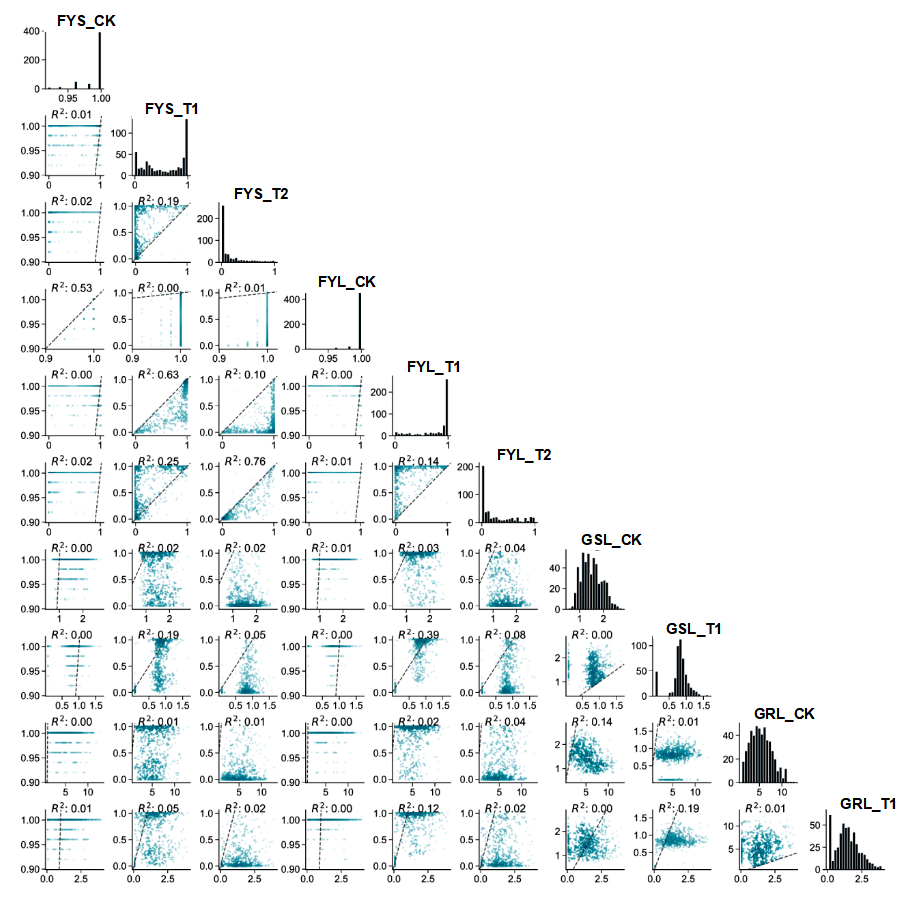


**Supplementary Figure S3.** Frequency distribution of all traits at germination stage.


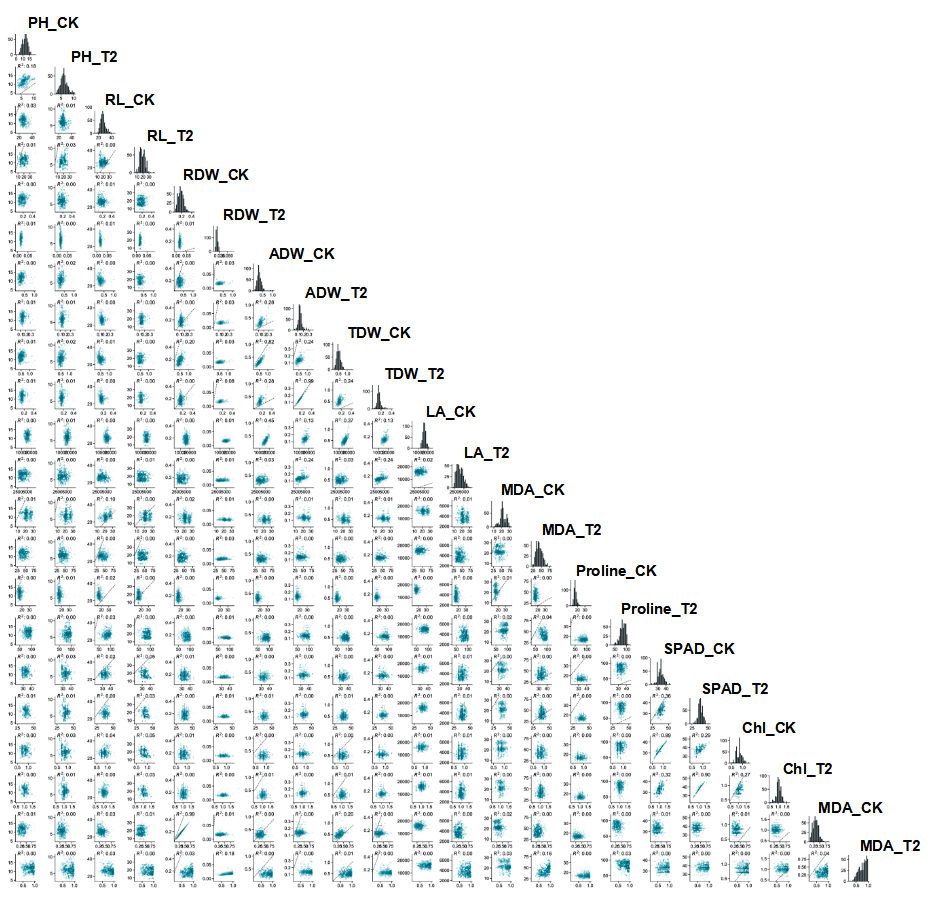


**Supplementary Figure S4.** Frequency distribution of all traits at seedling stage.


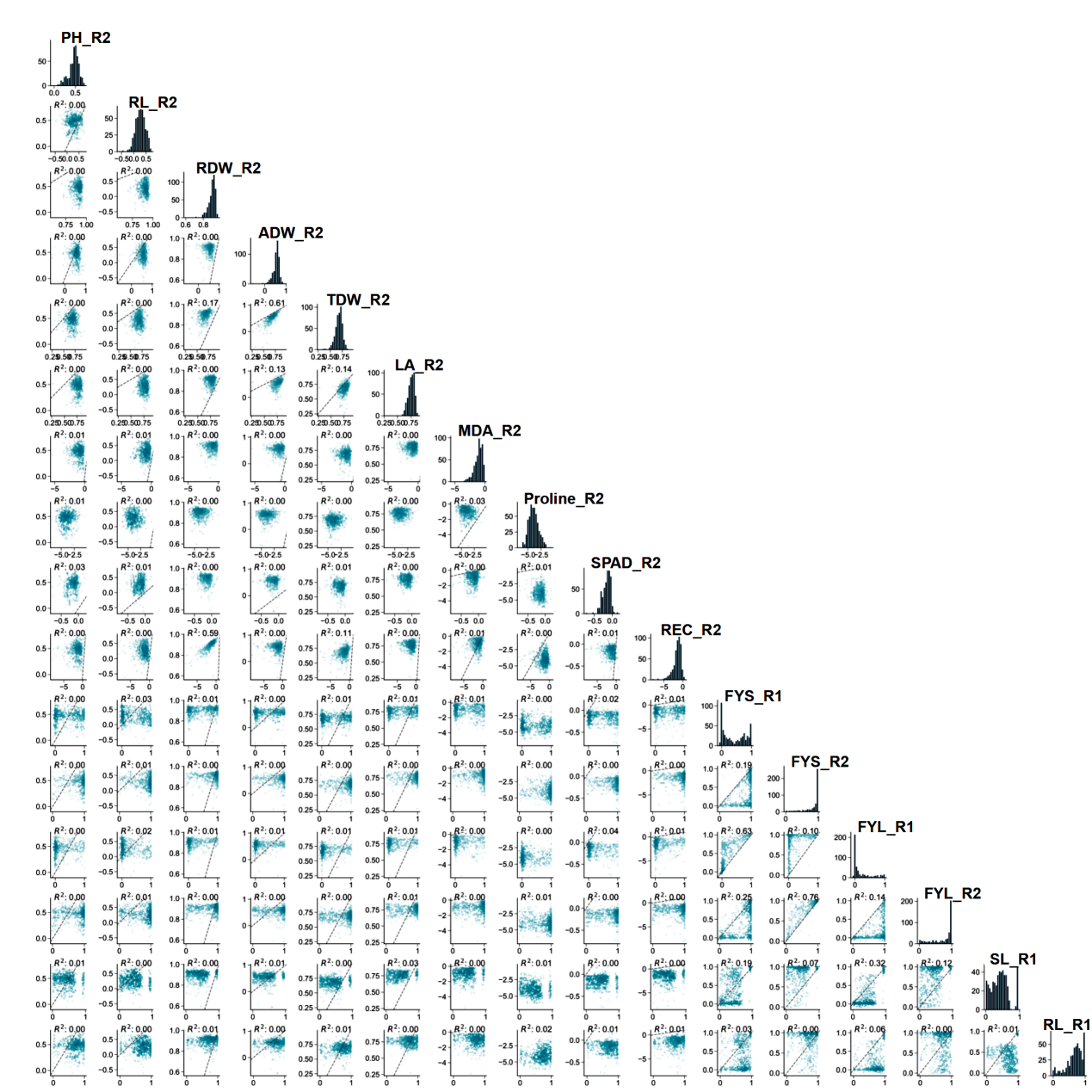


**Supplementary Figure S5.** Frequency distribution of STCs for GWAS at germination and seedling stages.


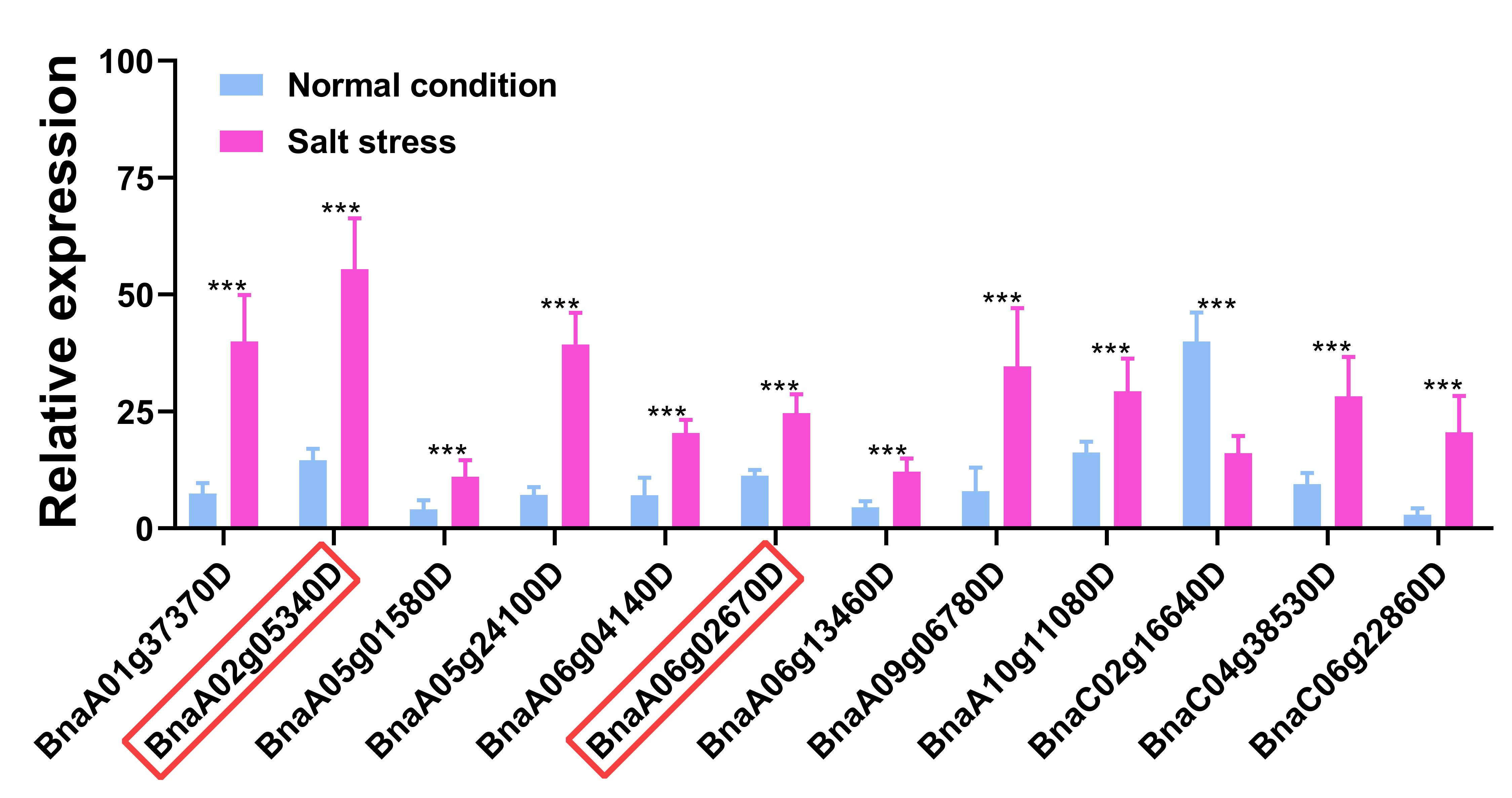


**Supplementary Figure S6**. RNA-seq based expression analysis of candidate genes under normal and salt stress conditions.


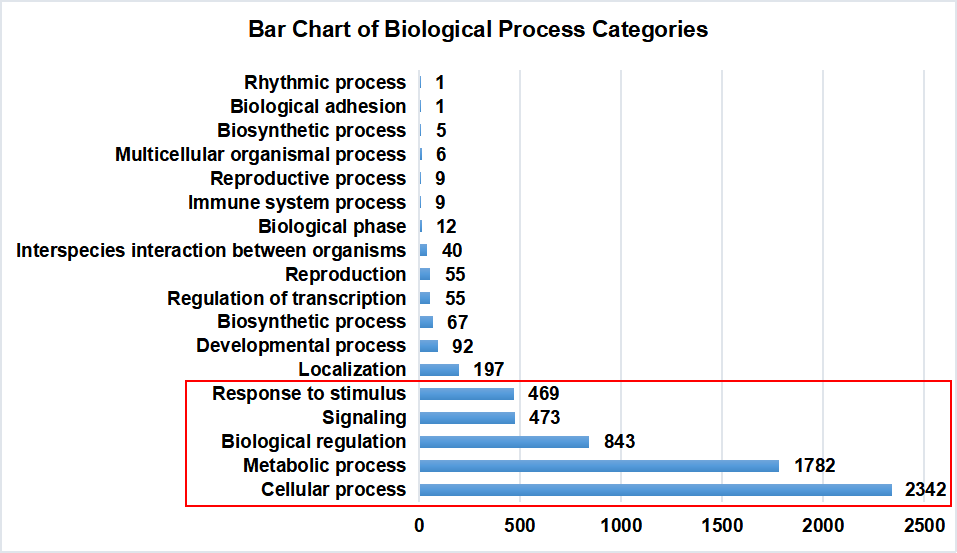


**Supplementary Figure S7.** GO enrichment analysis of candidate genes of all traits.


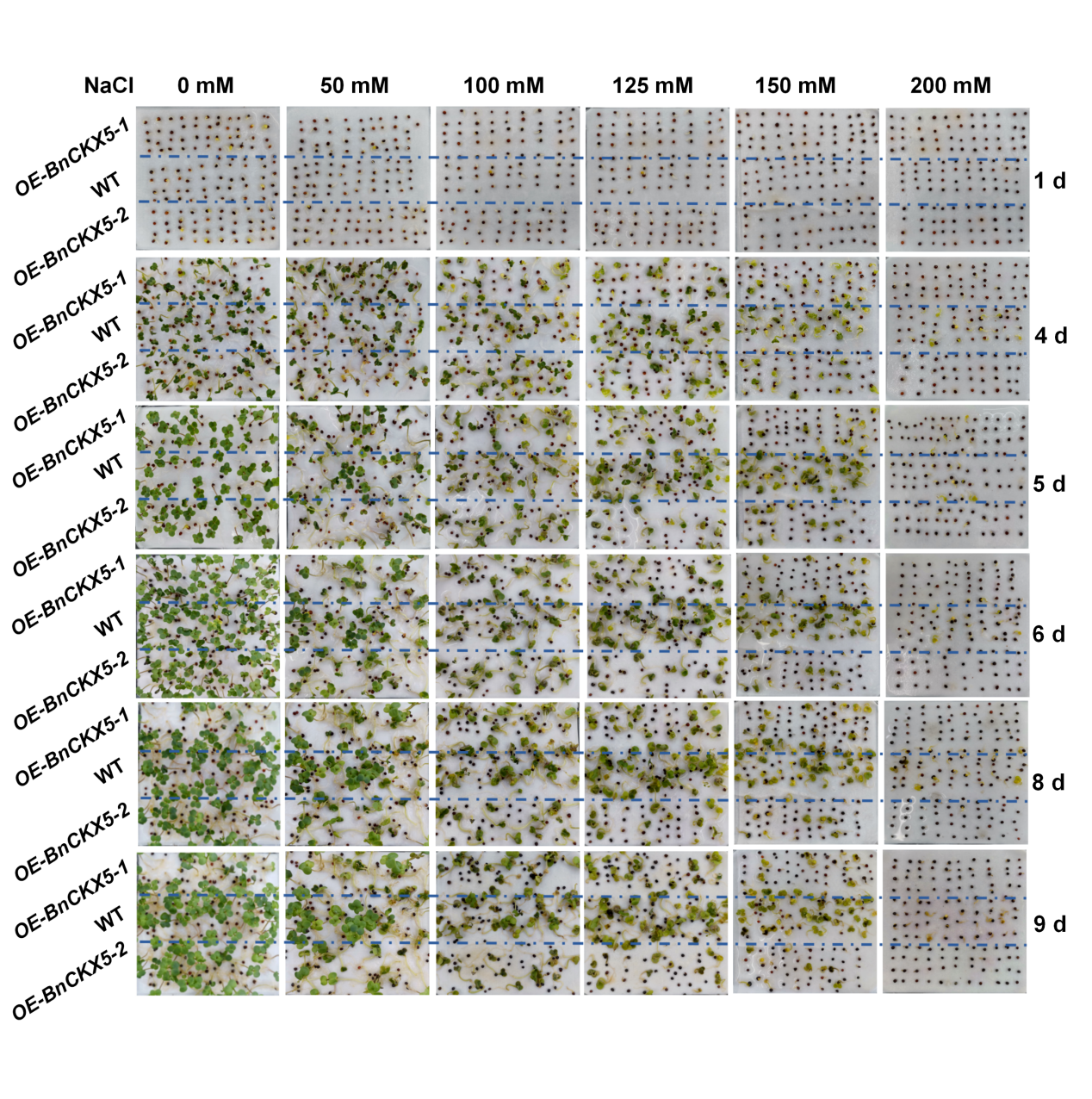


**Supplementary Figure S8.** *OE-BnCKX5* and WT plants grown under salt treatments at germination stage.


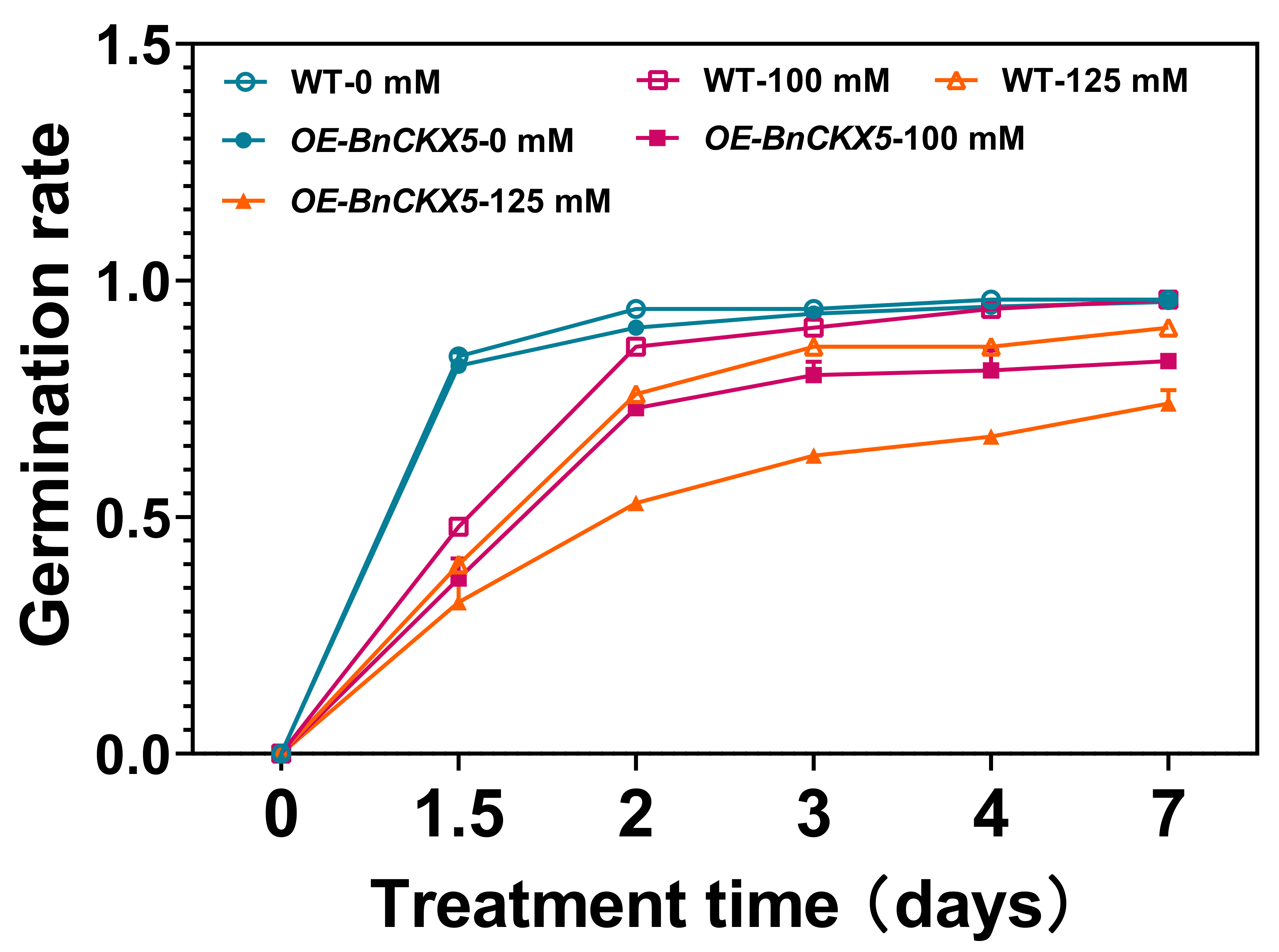


**Supplementary Figure S9.** Comparison of germination rate of *OE-BnCKX5* and WT under 0 mM, 100 mM and 125 mM NaCl.


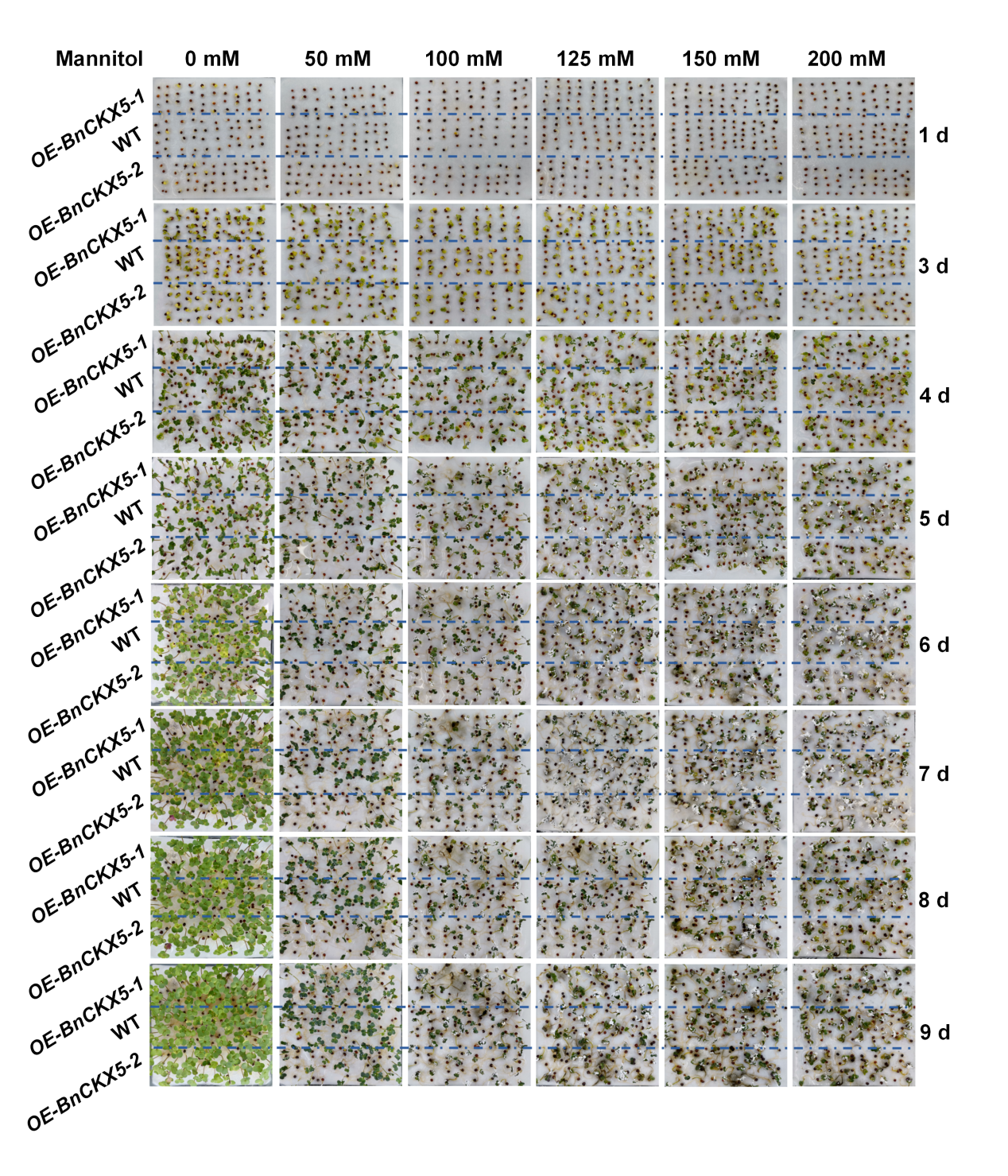


**Supplementary Figure S10.** *OE-BnCKX5* and WT plants grown under mannitol treatments at germination stage.


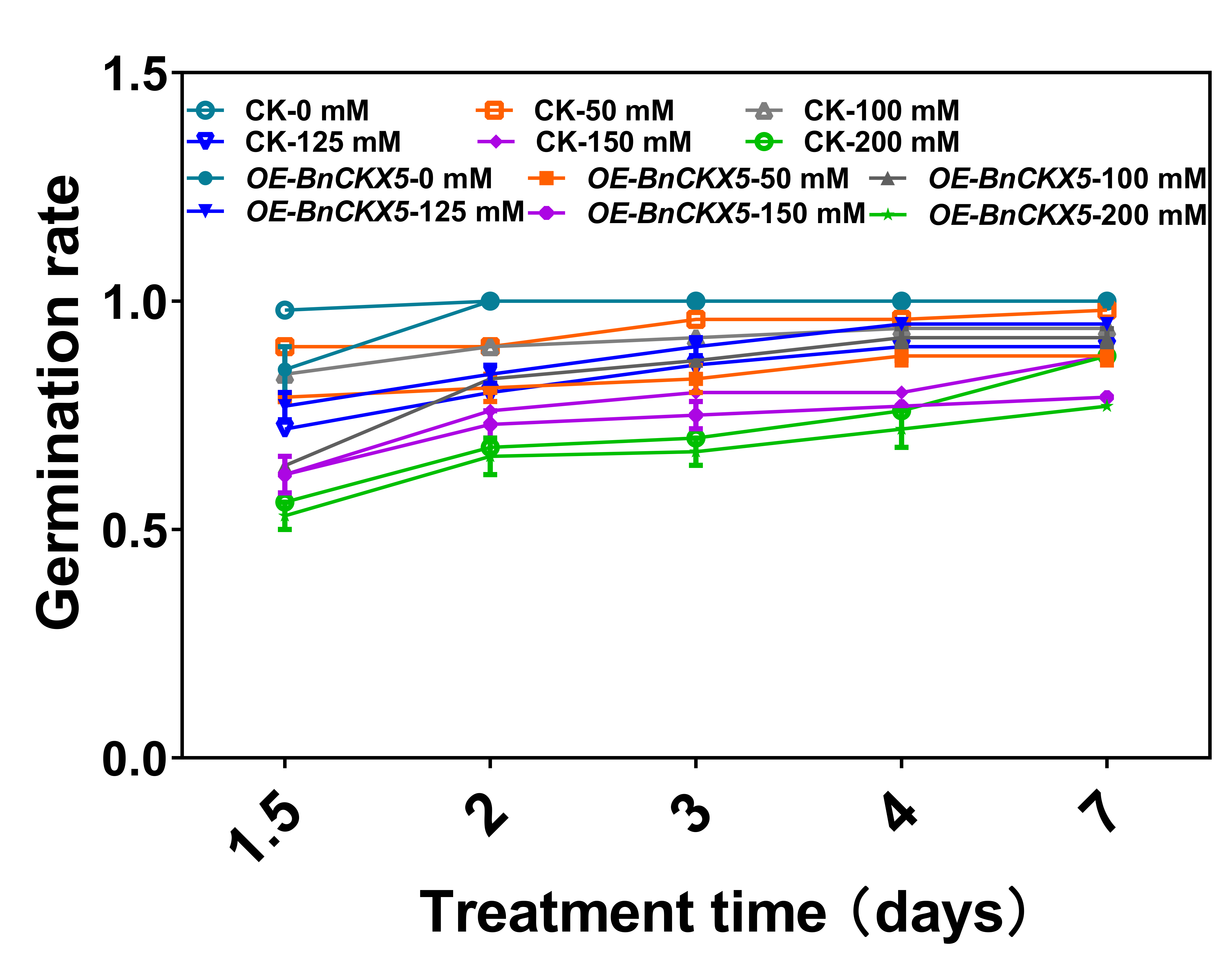


**Supplementary Figure S11.** Comparison of germination rate of *OE-BnCKX5* and WT under mannitol treatments at germination stage.


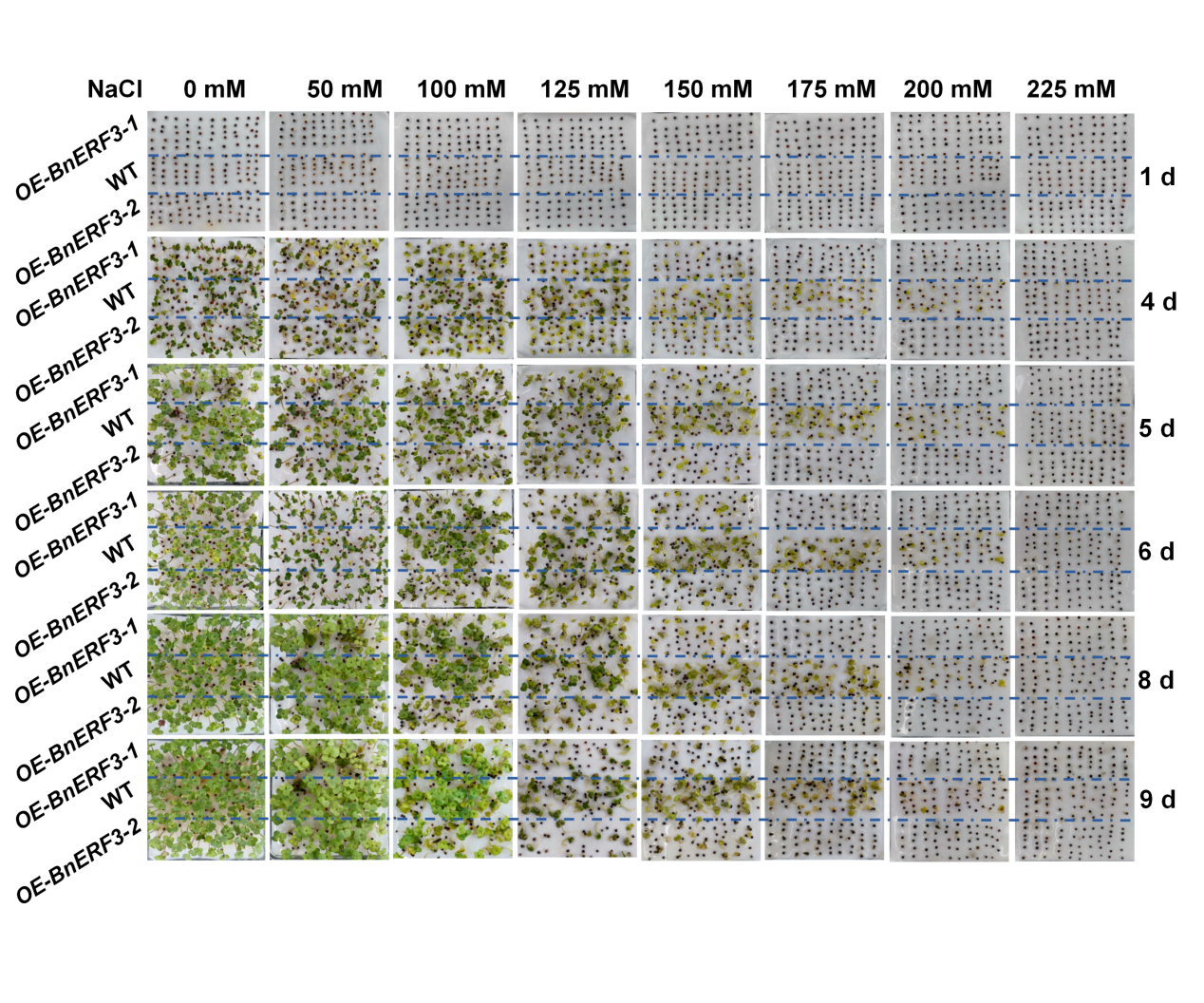


**Supplementary Figure S12.** *OE-BnERF3* and WT plants grown under salt treatments at germination stage.


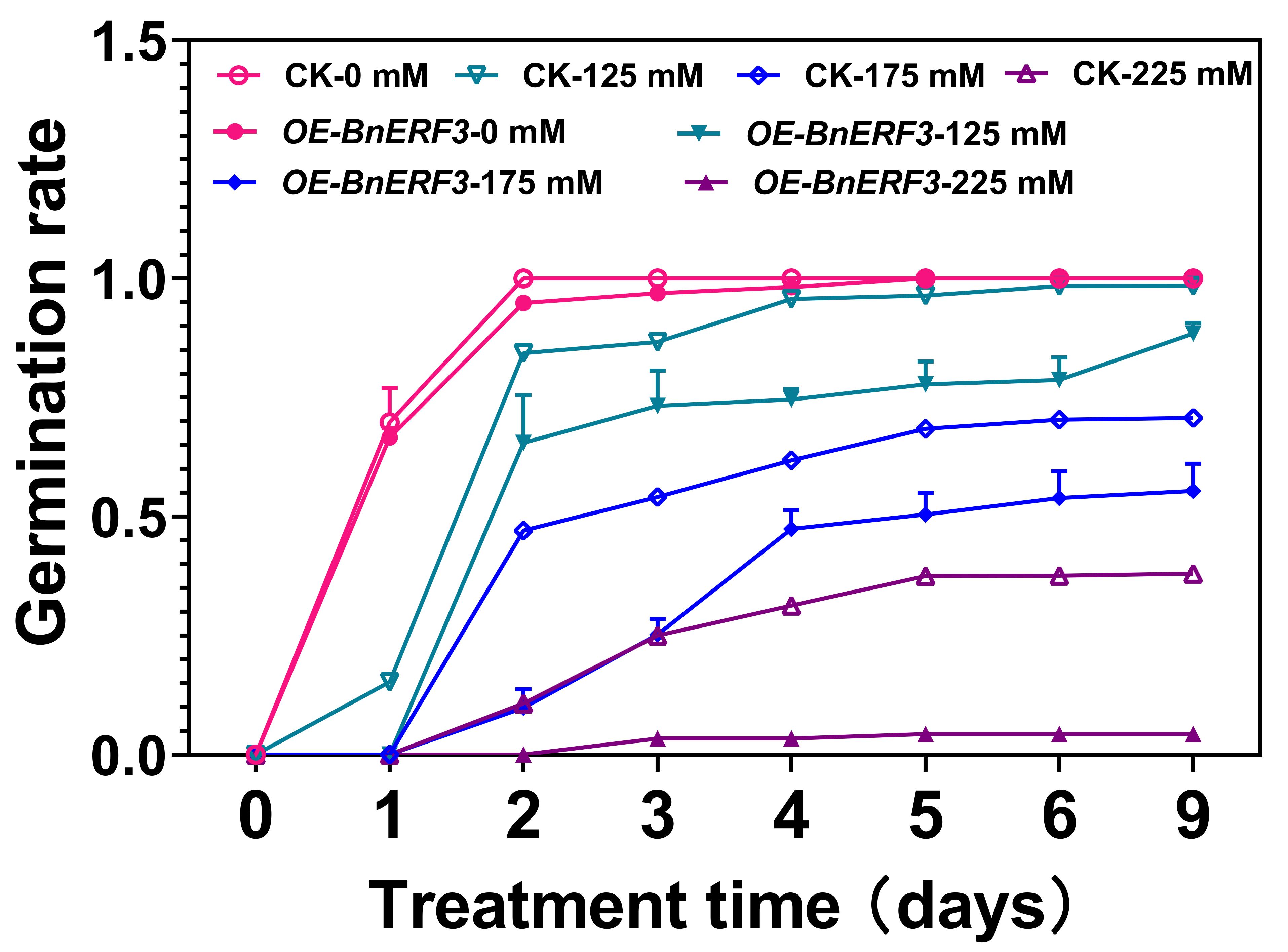


**Supplementary Figure S13.** Comparison of germination rate of *OE-BnERF3* and WT under 0 mM, 125 mM, 175mM and 225 mM NaCl at germination stage.


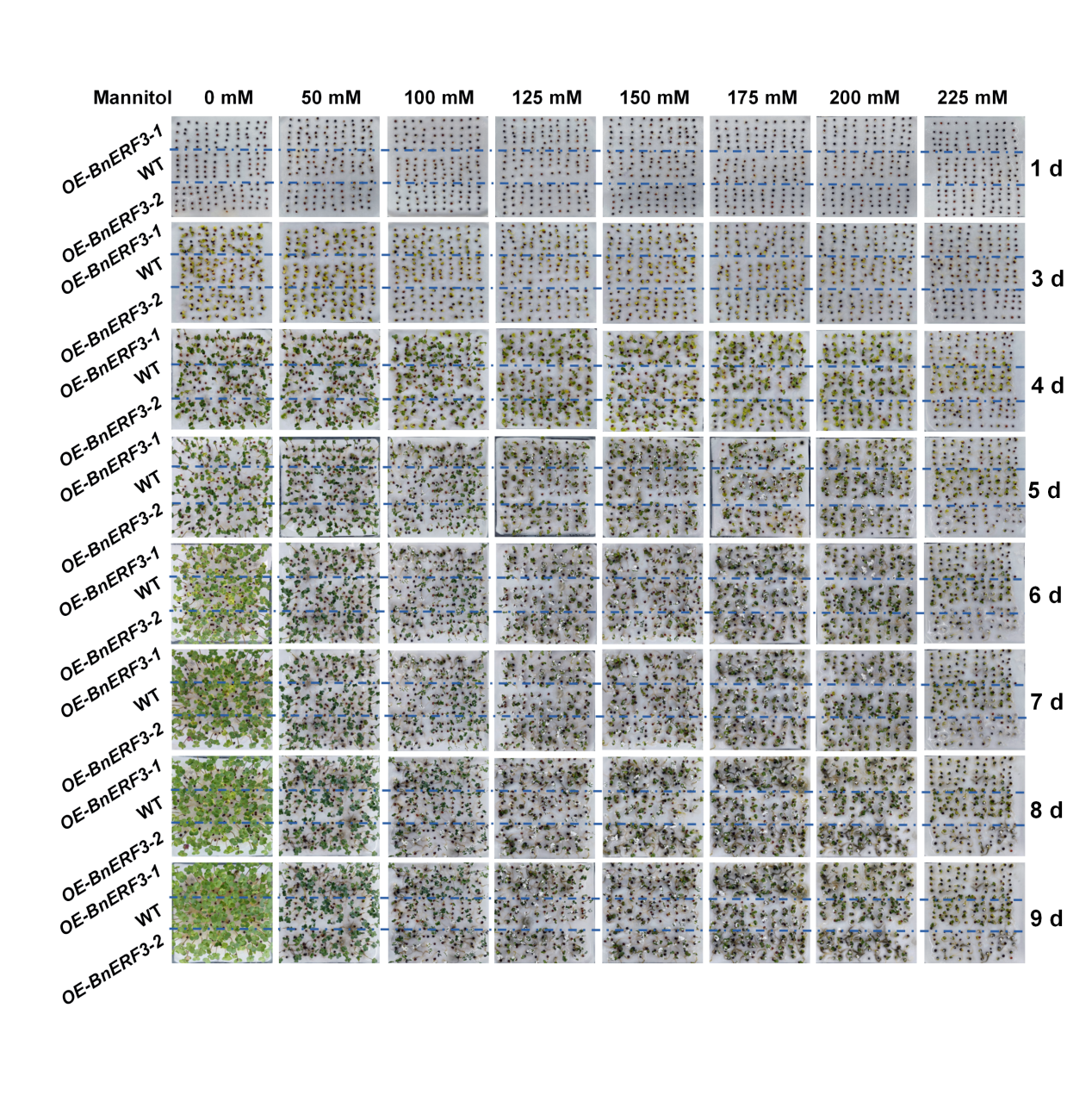


**Supplementary Figure S14.** *OE-BnERF3* and WT plants grown under mannitol treatments at germination stage.


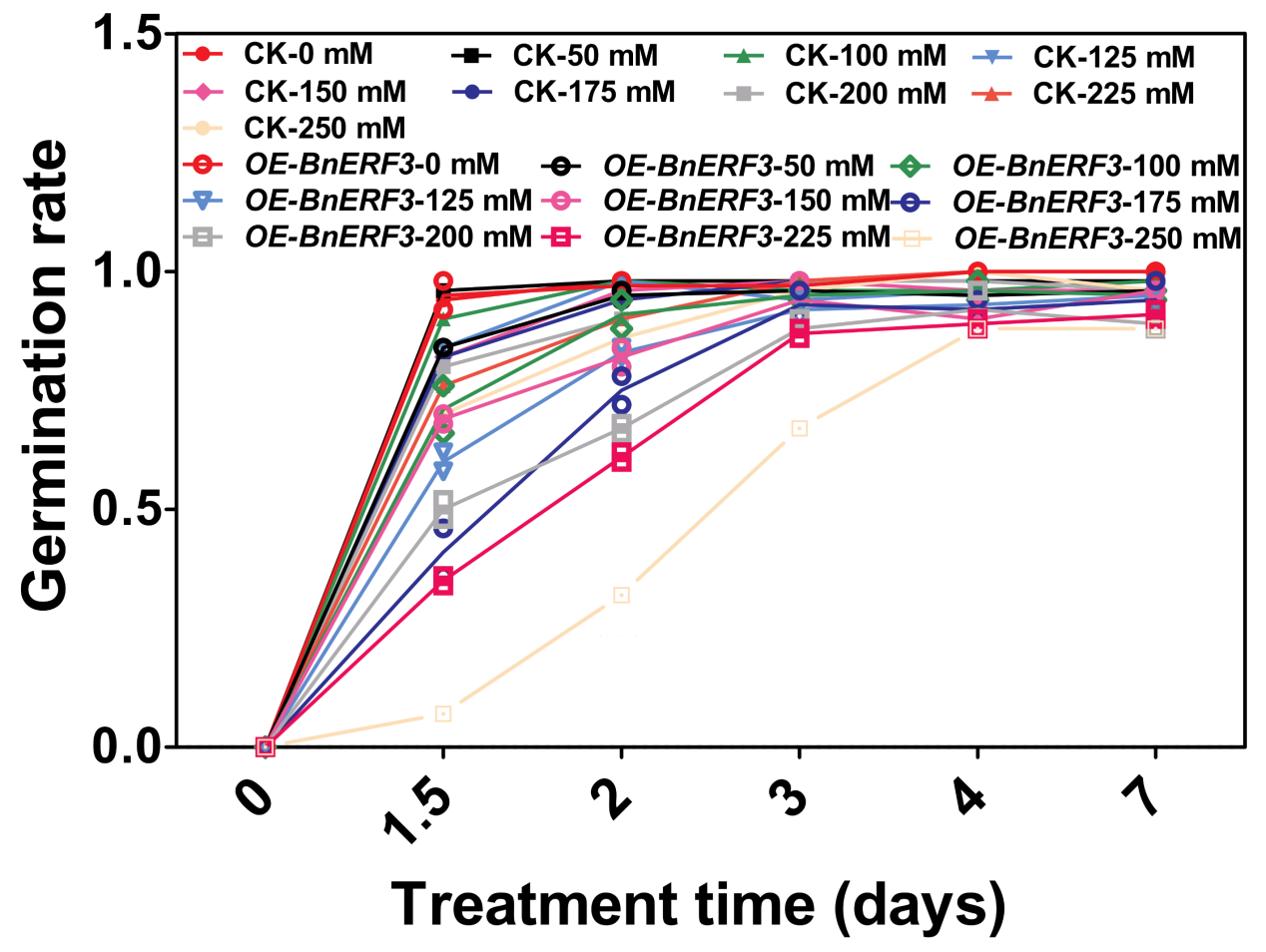


**Supplementary Figure S15.** Comparison of germination rate of *OE-BnERF3* and WT under mannitol treatments at germination stage.
